# Lasmiditan mechanism of action – review of a selective 5-HT_1F_ agonist

**DOI:** 10.1186/s10194-020-01132-3

**Published:** 2020-06-10

**Authors:** David B. Clemow, Kirk W. Johnson, Helen M. Hochstetler, Michael H. Ossipov, Ann M. Hake, Andrew M. Blumenfeld

**Affiliations:** 1 Eli Lilly and Company, Indianapolis, IN USA; 2 Evidera, Morrisville, NC USA; 3 Department of Neurology, Indiana University School of Medicine, Indianapolis, IN USA; 4 The Headache Center of Southern California, San Diego, CA USA

**Keywords:** Migraine, Lasmiditan, Lipophilicity, Brain penetration, 5-HT_1F_, CGRP, Glutamate

## Abstract

Migraine is a leading cause of disability worldwide, but it is still underdiagnosed and undertreated. Research on the pathophysiology of this neurological disease led to the discovery that calcitonin gene-related peptide (CGRP) is a key neuropeptide involved in pain signaling during a migraine attack. CGRP-mediated neuronal sensitization and glutamate-based second- and third-order neuronal signaling may be an important component involved in migraine pain. The activation of several serotonergic receptor subtypes can block the release of CGRP, other neuropeptides, and neurotransmitters, and can relieve the symptoms of migraine. Triptans were the first therapeutics developed for the treatment of migraine, working through serotonin 5-HT_1B/1D_ receptors. The discovery that the serotonin 1F (5-HT_1F_) receptor was expressed in the human trigeminal ganglion suggested that this receptor subtype may have a role in the treatment of migraine. The 5-HT_1F_ receptor is found on terminals and cell bodies of trigeminal ganglion neurons and can modulate the release of CGRP from these nerves. Unlike 5-HT_1B_ receptors, the activation of 5-HT_1F_ receptors does not cause vasoconstriction.

The potency of different serotonergic agonists towards 5-HT_1F_ was correlated in an animal model of migraine (dural plasma protein extravasation model) leading to the development of lasmiditan. Lasmiditan is a newly approved acute treatment for migraine in the United States and is a lipophilic, highly selective 5-HT_1F_ agonist that can cross the blood-brain barrier and act at peripheral nervous system (PNS) and central nervous system (CNS) sites.

Lasmiditan activation of CNS-located 5-HT_1F_ receptors (e.g., in the trigeminal nucleus caudalis) could potentially block the release of CGRP and the neurotransmitter glutamate, thus preventing and possibly reversing the development of central sensitization. Activation of 5-HT_1F_ receptors in the thalamus can block secondary central sensitization of this region, which is associated with progression of migraine and extracephalic cutaneous allodynia. The 5-HT_1F_ receptors are also elements of descending pain modulation, presenting another site where lasmiditan may alleviate migraine. There is emerging evidence that mitochondrial dysfunction might be implicated in the pathophysiology of migraine, and that 5-HT_1F_ receptors can promote mitochondrial biogenesis. While the exact mechanism is unknown, evidence suggests that lasmiditan can alleviate migraine through 5-HT_1F_ agonist activity that leads to inhibition of neuropeptide and neurotransmitter release and inhibition of PNS trigeminovascular and CNS pain signaling pathways.

## Background

Migraine is a painful, disabling neurological disease that has long afflicted the population worldwide. It was only in the past few decades that inroads have been made into understanding the pathophysiology of migraine and that specific treatments for migraine were developed [1]. The vascular theory of migraine held that the disorder was caused by vasodilation of meningeal arteries [1]. Based on this prevailing viewpoint, the prototypic triptan, sumatriptan, was initially developed to treat migraine due to its vasoconstrictive properties. The clinical success of sumatriptan led to the introduction of second-generation triptans and spurred an increased research effort into understanding the pathophysiology of migraine.

**Fig. 1:**
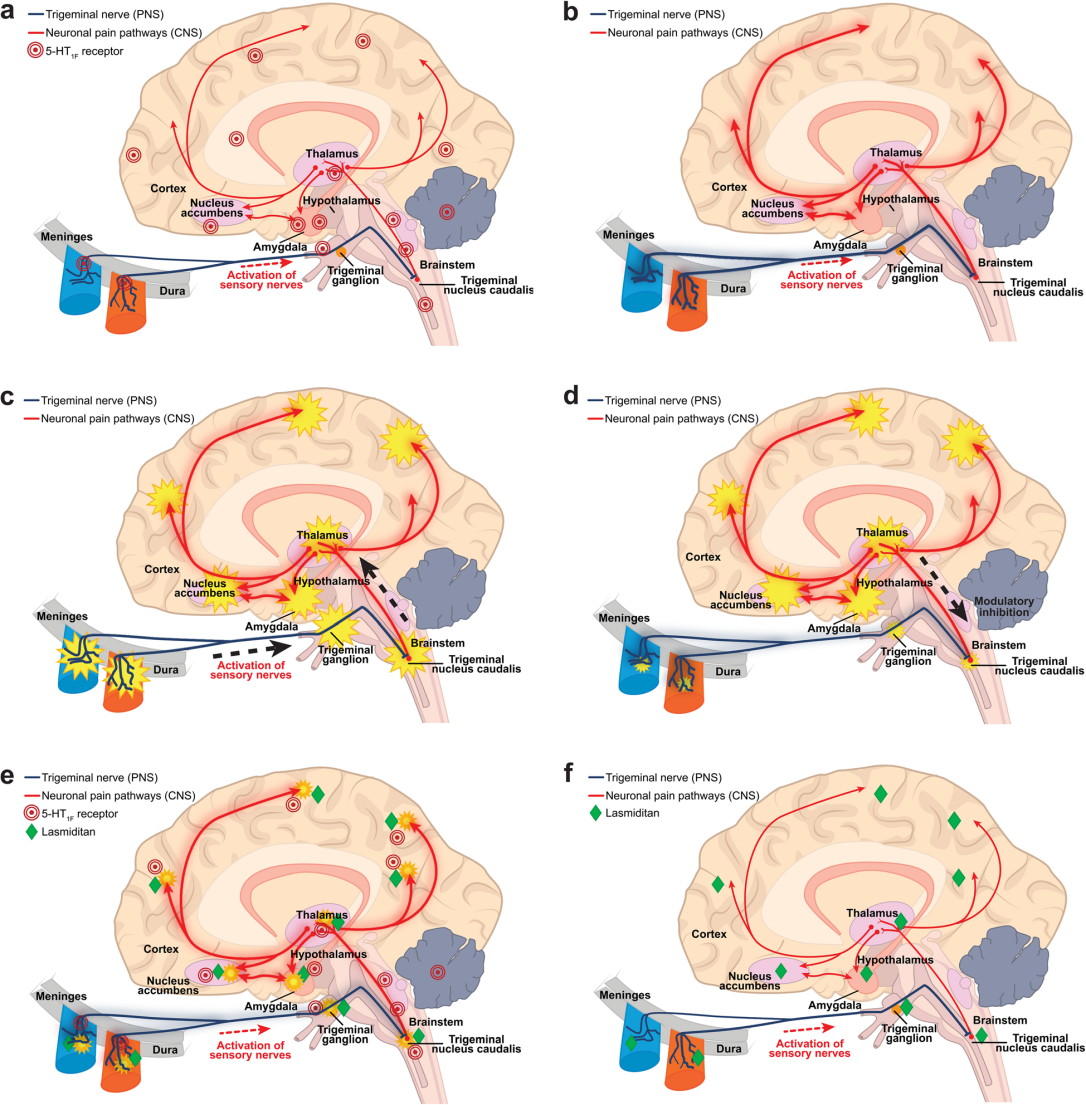
**a** Trigeminal and pain pathways associated with pain signaling during a migraine attack plus 5-HT1F receptor locations in PNS and CNS areas involved in the pathophysiology of migraine. **b** Activation of trigeminal and pain pathways during a migraine attack; sustained or repeated activation of pain pathways leads to increasing sensitivity of trigeminal neurons in the brain stem. **c** Activated nerves release various substances including neuropeptides and neurotransmitters such as CGRP and glutamate that can exacerbate neurogenic inflammation and nociceptor pain signaling in migraine; this may hyperexcite neurons to propagate pain responses and enhance central sensitization. **d** In addition to other endogenous inhibitory processes, serotonin from pain modulating pathways activates 5-HT receptors that can inhibit neuropeptide and neurotransmitter release, thereby inhibiting their local activity and downstream neuronal signaling; however, these modulatory mechanisms may be disturbed in migraine pathophysiology, predisposing patients with migraine to an attack. **e** Lasmiditan crosses the blood-brain barrier and presumably acts in both the PNS and the CNS to selectively activate 5-HT1F receptors to inhibit the release of neuropeptides and neurotransmitters such as CGRP and glutamate, thereby inhibiting their local activity and migraine attack pain pathways. **f** Preclinical and ex vivo human evidence suggests that lasmiditan inhibits PNS trigeminal nerve and CNS pain signaling pathways, exerting therapeutic effects in the treatment of migraine without causing vasoconstriction. Abbreviations: CNS, central nervous system; mRNA, messenger ribonucleic acid; PNS, peripheral nervous system. Note: 5-HT1F receptor locations are based on human and animal mRNA, immunohistochemistry, and functional biology studies

**Fig. 2:**
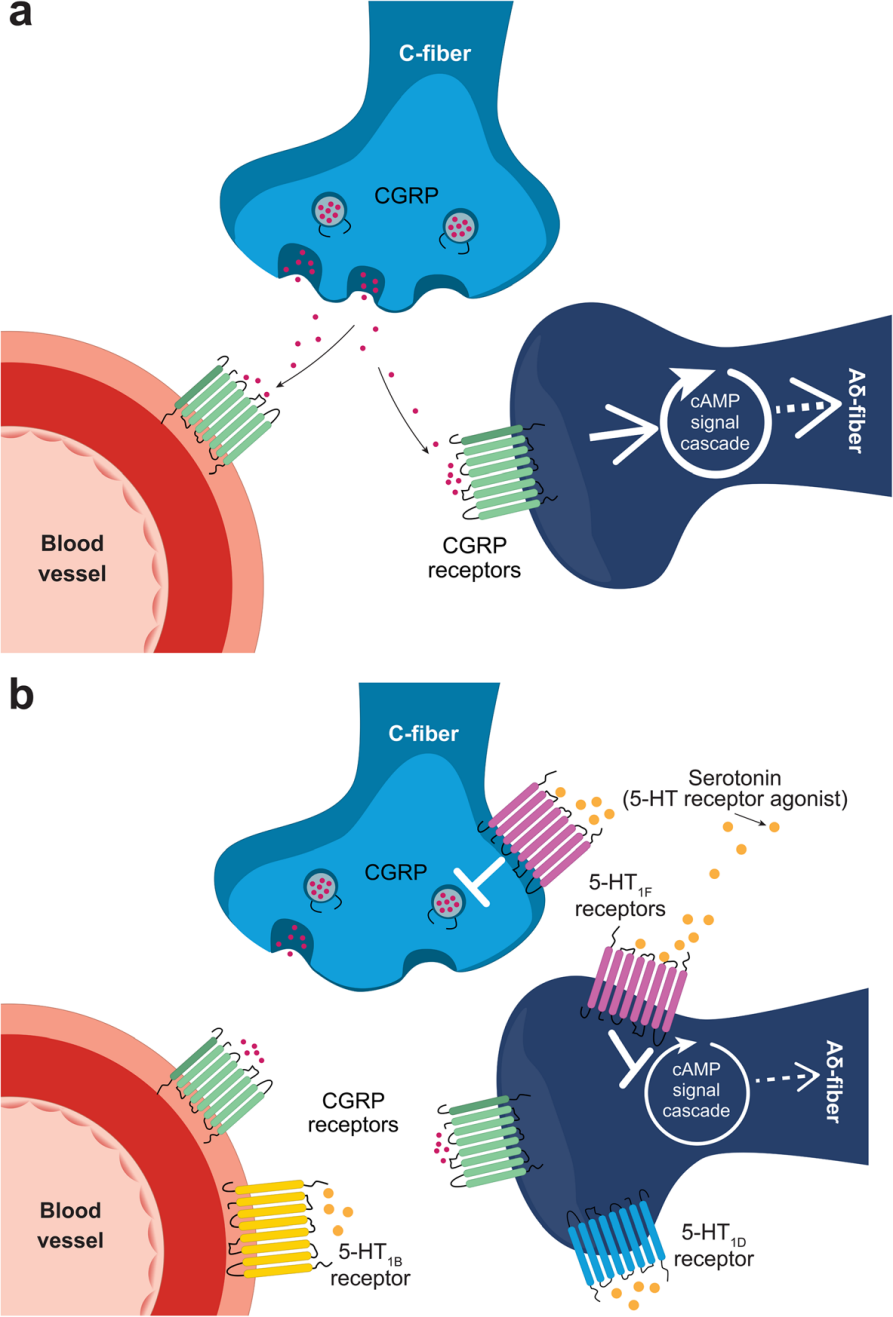
**a** Neuropeptides and neurotransmitters regulate pain during a migraine attack. Nerve activity in the trigeminovascular pain pathway leads to the release of neuropeptides and neurotransmitters, such as CGRP and glutamate that can hyperexcite neurons, thereby propagating PNS and CNS pain responses. **b** The thalamic trigeminovascular neurons, trigeminal ganglion, and the trigeminal nucleus caudalis are densely innervated with serotonergic neurons. Subtypes of serotonin (5-HT) receptors found in these areas, such as 5-HT1F, are believed to be involved in the pathophysiology of migraine. Serotonin receptor agonists are the foundation of many acute treatments for migraine; however, many of these therapeutics are associated with vasoconstriction through activation of 5-HT1B receptors. Serotonin binding to 5-HT1F receptors can inhibit presynaptic vesicular release of CGRP and inhibit postsynaptic cAMP signaling cascades. Abbreviations: 5-HT, 5-hydroxytryptamine (serotonin); 5-HT1, 5-hydroxytryptamine 1 receptor; cAMP, cyclic adenosine monophosphate; CGRP, calcitonin gene-related peptide; CNS, central nervous system; PNS, peripheral nervous system


Numerous studies have since provided convincing evidence that migraine is a neurological disease with a prominent role in the activation of the trigeminovascular system (Fig. 1a, Supplemental Video). It was also determined that calcitonin gene-related peptide (CGRP), a neuropeptide prominently expressed in trigeminal afferent neurons, plays a key role in the neurophysiology of migraine (Fig. 2a, Supplemental Video) [2–4]. Research has shown that the triptans exert their antimigraine effects by blocking the release of CGRP and potentially other neuropeptides from trigeminal afferent fibers. The triptans appear to work through activation of the serotonergic 5-HT_1B/D_ receptors rather than vasoconstriction [2, 3, 5]. The vasoconstrictive effect is mediated through the 5-HT_1B_ receptor [5].

**Additional file 1.**



Triptans are generally considered safe overall, but they are contraindicated in patients with uncontrolled hypertension and other cardiovascular and cerebrovascular diseases [6]. The triptans vary somewhat in their ability to cross the blood-brain barrier (BBB). Data suggests that overall, the triptans seem to remain largely restricted to peripheral nervous system (PNS) sites of action [6]. However, recent evidence in rats suggests that subcutaneous sumatriptan can accumulate rapidly in the central nervous system (CNS) [7, 8]. The development and clinical efficacy of non-vasoconstriction drugs for the treatment of migraine led to the conclusion and current predominate theory that vasoconstriction is not the triptan’s therapeutic mechanism of action. Attempts to develop 5-HT_1D_ agonists to treat migraine were largely unsuccessful; and a newly recognized class of drugs, the “ditans,” was developed to selectively act at the 5-HT_1F_ receptor.

With its approval in October 2019 in the United States, lasmiditan is currently the only selective 5-HT_1F_ agonist approved for treatment of acute migraine attack. Lasmiditan is considered a first-in-class “ditan” based upon its unique chemical structure with a pyridinoylpiperidine scaffold structure, 2,4,6-trifluoro-N-[6-(1-methylpiperidine-4-carbonyl)pyridine-2-yl] benzamide hemisuccinate, and its selective high affinity for the 5-HT_1F_ receptor. Preclinical studies showed that lasmiditan is devoid of vasoconstrictive effects [9] and that it penetrates the BBB into the CNS [10], which is important since the CNS also plays a role in the management of migraine [11]. Lasmiditan has shown efficacy in the acute treatment of migraine in randomized, double-blind, placebo-controlled clinical trials (RCT) [12–15]. This narrative review highlights the pharmacology and pharmacokinetics of lasmiditan and its pharmacological activity at the 5-HT_1F_ receptor as well as the role of the 5-HT_1F_ receptor as a potential mechanism to be exploited in treating migraine.

This review discusses the potential mechanism of action for lasmiditan in the context of overall migraine pathophysiology. Data supports that migraine pathophysiology involves activation of the hypothalamus, producing the prodrome phase premonitory symptoms of migraine that are followed by trigeminal ganglion (TG) activation, which leads to the headache phase [16]. Trigeminal system activation results in the release of multiple neuropeptides, including CGRP, pituitary adenylate cyclase-activating polypeptide (PACAP), and substance P, at both the PNS and CNS terminals of the trigeminal nerve [17]. CGRP has been shown to be involved in migraine [2]. Preclinical and clinical studies failed to support a role for substance P in migraine. Although substance P and CGRP are co-localized in pre-synaptic vesicles and released together [18], trials assessing agents that block only substance P have not shown benefit in the treatment of migraine [19, 20]. Conversely, emerging evidence suggests that PACAP may contribute to migraine [17, 21].

Afferent activation of the trigemino-thalamic pathway and sensory cortex leads to sensitization and allodynia, which can lead to chronification – progression in frequency of migraine attacks [16, 22]. The CNS afferent pathways involve the excitatory neurotransmitter glutamate. The ascending CNS pain pathways are modulated by descending pain modulatory pathways that include projections to the periaqueductal gray matter (PAG), noradrenergic locus coeruleus, and the nucleus raphe magnus (NRM). The descending pathways relay serotonergic and non-serotonergic pain inhibitory and facilitatory neural projections [23–27]. Inhibitory projections can attenuate pain signaling likely in part by activating 5-HT_1F_ receptors on glutamate-containing neurons [3, 24]. These anatomic and neuronal signaling pathways could be potential neurological points of activity for ditan therapy.

### Literature search methods

The PubMed, Embase, and Cochrane Library databases were searched for the terms “LY573144, lasmiditan, OR LY334370 OR (COL-144 OR COL144 OR COL 144).” The terms included variations of the “LY” numbers to include hyphens, commas, or spaces, and LY334370 was included since it was a 5-HT_1F_ receptor agonist that preceded lasmiditan in development. A separate search was conducted for “(5-HT OR serotonin) AND (mechanism OR mechanism of action) AND (blood brain barrier) AND (central penetrant) AND (physiology OR pathophysiology OR pharmacology) AND (lasmiditan OR ditan).” Results were examined to identify primary sources that address the basic pharmacology of 5-HT_1F_ agonists or their potential role in the PNS and CNS in treating migraine. Review articles published in 2018 and 2019 were examined to find any primary sources that may have been missed in the searches. Review articles are included in this manuscript to support ancillary points, to support tangential facts, and to buttress arguments. This is a comprehensive and balanced overview of the current understanding of the role of 5-HT_1F_ in migraine pathophysiology; therefore, all relevant studies may not be specifically referenced.

### PNS and CNS neuropathology of migraine

Research in the mid-1600s proposed that migraine was caused by meningeal vasodilation, a view that persisted through the 1900s [1]. An alternate theory that migraine arose from “nerve storms evolved out of the optic thalamus,” was put forth in 1783 [1]. The current working hypothesis of migraine is that it is a complex neurological disease that is mediated through the trigeminovascular system (Fig. 1b, Supplemental Video) [28–30]. Some studies that correlated observations made in animal models with clinical observations, including imaging studies, suggested that the headache phase of a migraine attack corresponds to the development of peripheral sensitization involving the primary afferents from the TG (Fig. 1c, Supplemental Video) [31–33]. As the attack progresses, central sensitization of the trigeminal nucleus caudalis (TNC) occurs, with enhanced responses of nociceptive TNC neurons, and expansion of receptive fields to facial areas. The TNC has extensive connections with other brain regions. Prolonged activation of the TNC results in sensitization of third-order neurons in the thalamus, which can account for the expansion of cutaneous allodynia to extracephalic regions [31, 33–36]. The TNC also has reciprocal connections with brain regions that are implicated in pain processing, such as the insula, PAG, and the rostral ventromedial medulla [37]. Further progression of central sensitization results in sensitization of thalamic third-order neurons receiving inputs from the TNC; this is associated with the spreading of cutaneous allodynia to extracephalic regions [31, 33]. For example, enhanced activation of the posterior thalamus in response to light cutaneous stimuli applied to the dorsum of the hand of a patient during a migraine attack was demonstrated in one functional magnetic resonance imaging (fMRI) study. The shift from peripheral sensitization to central sensitization may be demonstrated by the coincident loss of efficacy of triptans in reversing a migraine headache as the migraine attack progresses, as many of these therapeutics generally do not appear to act centrally [38].

Recent reviews suggested that the genesis of a migraine attack remains a mystery [24, 25, 39]. It has been proposed that cortical spreading depression (CSD), a wave of depolarization and repolarization across the cortex, may trigger neurogenic inflammation, with plasma extravasation and mast cell degranulation. Previous speculations have associated migraine with disruption of the BBB [40]; however, more recent clinical observations showed that the BBB remains intact during a migraine attack [41, 42]. Animal models were developed to study these ideas, but it is unclear to what extent they represent the clinical situation. CGRP can degranulate mast cells in rats, but human mast cells do not have functional CGRP receptors and are not responsive to CGRP [43]. Moreover, spontaneous CSD has not been demonstrated in humans, and there are no studies proving that CSD leads to a migraine attack [44].

Imaging studies showed activation of the hypothalamic areas when migraine is provoked by injections of nitroglycerin or CGRP that produce the premonitory symptoms of the prodrome phase before the headache starts. This is consistent with premonitory symptoms such as food craving, fatigue, nausea, and yawning which suggest hypothalamic involvement in migraine [16, 45]. Studies using H_2_^15^O positron emission tomography (PET) cerebral blood flow scans showed increased hypothalamic activity during the early prodrome phase in patients who had migraine induced with nitroglycerin [46]. Increased hypothalamic activity along with functional coupling to the TNC was detected by fMRI in a patient who experienced two to three migraine attacks per month and underwent daily scans for 30 days [47]. It is believed that oscillations in hypothalamic activity alter functional connections between the hypothalamus and brainstem, altering susceptibility thresholds to sensory stimuli thereby initiating and ending a migraine attack [48]. Another view [24, 45] suggested that cortical excitability influences activity at other sites, including pain modulatory systems, to initiate migraine. A central locus for the initiation of migraine is consistent with the fact that the premonitory symptoms that precede headache can occur many hours before the pain. Peripheral sensitization can develop as a result of enhanced CNS activity through as yet undetermined mechanisms, but possibly involving oscillations in hypothalamic activity, eventually resulting in migraine headache pain [16, 24, 45, 48]. These data point to the importance of the CNS as a potential target tissue for migraine therapy.

### Role of CGRP in migraine

During the initial phase of a migraine attack, CGRP is released from trigeminal afferent C-fibers that are bi-directional. This released CGRP can activate the CGRP receptors on adjacent trigeminal afferent Aδ fibers that are bi-directional and project back to the TNC (Fig. 2a). While CGRP does not produce nociception, it can promote central sensitization of the TNC second-order neurons. CGRP released in the PNS can dilate the meningeal arteries due to its potent vasodilatory effect, but it also activates signaling cascades that produce nitric oxide (NO) and release glutamate and prostaglandins from the arterial wall. These substances can sensitize and/or activate the trigeminal afferent fibers of the PNS, leading to enhanced release of CGRP and promoting a feed-forward enhancement mechanism. Activation of these PNS nociceptors is an important step in the early phase of the migraine headache [2, 24, 25].

Continued activation of the primary afferents in the TNC, together with the release of CGRP, as well as glutamate and possibly other neurotransmitters, promotes sensitization of these second-order neurons [49–51]. Central sensitization, driven in large part by the excitatory neurotransmitter glutamate, is associated with reduced activation thresholds, increased firing in response to inputs, continued after-discharge in response to inputs, recruitment of adjacent neurons, and expansion of their fields [31, 33–36, 49, 51]. This mechanism can account for the development of cutaneous allodynia and its expansion in facial regions. Whereas the role of CGRP in the trigeminovascular system is well examined regarding migraine, how or whether CGRP is instrumental at CNS sites requires further investigation.

The small molecule CGRP antagonist, telcagepant, does not appear to act centrally at therapeutic doses [52]. The small molecule GGRP receptor antagonists such as ubrogepant and rimegepant, which were recently approved in the United States for the treatment of migraine attacks [53, 54], appear to predominantly function in the PNS [24, 55]. While the BBB is relatively impermeable to antibodies [56], it is still possible to detect up to approximately 0.1% of the blood concentration in the cerebrospinal fluid [24, 57, 58]; thus, the monoclonal antibodies to CGRP peptide (eptinezumab, fremanezumab, and galcanezumab) could potentially have some level of CNS activity, despite not appearing to penetrate the BBB in large concentrations [24, 57]. These data suggest that there is a need for migraine therapeutics that could act in the CNS. Importantly, the activity of CGRP at CNS sites (e.g., amygdala, PAG, parabrachial nucleus, and NRM) can be either pronociceptive or antinociceptive, depending on the brain region involved, suggesting that the role of CNS CGRP in migraine may be complex [24, 25, 59].

### 5-HT_1F_ receptor location

The human 5-HT_1F_ receptor was first identified and cloned in 1993 [60]. Messenger RNA (mRNA) for the 5-HT_1F_ receptor was found in human brain, uterine, and mesenteric tissue, but not in heart, kidney, liver, pancreas, spleen, or testes. In situ hybridization showed the presence of mRNA in the cerebral cortex, hippocampus, and dorsal raphe of guinea pig brains [60] and in the guinea pig and human TG [61–63]. In other studies, mRNA for 5-HT_1F_ was detected in human cerebral blood vessels [61], rat meningeal dura mater [64], cerebral cortex, and TG [65]. Moderate levels were detected in coronary and pulmonary blood vessels, but not in skeletal muscles or the mesenteric artery [65]. Studies performed with isolated human microvasculature detected 5-HT_1F_ message in microvessels and capillaries, but not in cultures of isolated human brain smooth muscle cells, suggesting that the 5-HT_1F_ receptors were present on astrocytes associated with the vessels [66].

Autoradiographic in situ hybridization studies performed with guinea pig brain sections showed the presence of mRNA for 5-HT_1F_ receptors in several regions. These included cortical and hippocampal sites, the claustrum, amygdala, the mediodorsal and laterodorsal thalamic nuclei, supraoptic hypothalamic nucleus, periventricular hypothalamic area, and several midbrain and medullary sites including the dorsal raphe, anterior pretectal area, ventral tegmental nucleus, paragigantocellular reticular nucleus, and the TNC [67].

Prior to the availability of selective ligands for 5-HT_1F_, triptans were used to visualize the distribution of 5-HT_1F_ receptors as some triptans bind to 5-HT_1F_ in addition to 5-HT_1B/D_. Autoradiographic studies with [^3^H]-sumatriptan, with and without 5-carboxamidotryptamin (5-CT) used to displace sumatriptan from 5-HT receptors, showed the presence of the 5-HT_1D_ and 5-HT_1F_ receptor subtypes in the human frontal cortex, globus pallidus, PAG, and TNC [68]. Autoradiography in rats and guinea pigs showed the presence of 5-HT_1F_ in the PAG, TNC, hippocampus, and cortex [69, 70]. Autoradiographic signals in the human brain were observed at high levels in the globus pallidus and substantia nigra, moderate levels in the fronto-temporal cortex, caudate-putamen and hippocampus, and low levels in the cerebellum, pons, medulla, and spinal cord [71].

LY334370 is a high affinity, selective agonist that acts at the 5-HT_1F_ receptor with approximately 300-fold greater selectivity compared to other 5-HT receptors [72] and is a useful pharmacologic tool for examining the 5-HT_1F_ receptor [72]. Thus, [^3^H]LY334370 was used for autoradiographic studies performed on guinea pig and rat brains, and preliminary studies (*N* = 1 each) with rhesus monkey and human brain sections [72]. In those studies, specific binding sites were detected in the cortex, amygdala, thalamus, nucleus accumbens, caudate putamen, hippocampal CA3 region, olfactory bulb, and tubercle of the rat, as well as in the cortex, caudate putamen, nucleus accumbens, thalamus, and medial mammillary nucleus of the guinea pig [72].

Based on currently described human and animal mRNA, immunohistochemistry, and functional biology studies, 5-HT_1F_ receptor locations are on neuronal synapses within PNS and CNS structures involved in the pathophysiology of migraine and structures modulating associated pain signaling, including but not limited to meninges, TG, trigeminal nucleus caudalis, hypothalamus, thalamus, and cortex (Fig. 1a) [72–76].

### Role of 5-HT_1F_ receptor in migraine

Nerve activity in the trigeminovascular pain pathway leads to the release of neuropeptides and neurotransmitters, such as CGRP and glutamate, and this activity is thought to exacerbate neurogenic inflammation and nociceptor pain signaling in migraine (Fig. 1c) [24, 77, 78]. The release of CGRP and glutamate can hyperexcite second-order neurons, which may propagate PNS and CNS pain responses [24, 77, 78]. The 5-HT_1F_ receptors are located on neuronal synapses involved in this modulating of pain signaling (Fig. 2b, Supplemental Video) [74, 75].

Dose-dependent inhibition of forskolin-induced cyclic adenosine monophosphate (cAMP) formation by 5-HT_1F_ agonists showed that the 5-HT_1F_ receptor, like other subtypes in the 5-HT_1_ family, is coupled to the G_i/o_ G-protein and, when activated, inhibits adenylate cyclase-mediated formation of cAMP (Fig. 2b). This inhibits the phosphorylation of protein kinase A (PKA) and affects downstream signaling pathways, including the inhibition of neuropeptide and neurotransmitter release [60]. Evidence suggests that serotonin activity at the 5-HT_1F_ receptor on neuronal synapses inhibits the release of CGRP and glutamate, reducing hyperexcitability and regulating pain signaling (Fig. 2b, Fig. 1d, Supplemental Video) [24, 77].

Immunohistochemical studies of cervical, thoracic, and lumbar dorsal root ganglia (DRG) and TG of rats with antibodies selective for 5-HT_1B_, 5-HT_1D_, or the 5-HT_1F_ receptors showed that the majority of DRG and TG neurons were labelled for each of the subtypes. Immunolabeling was noted on small-diameter C-fiber neurons, Aδ neurons, and the larger Aβ neurons [73]. Double-labelling immunohistochemistry of TG in rats found that the majority of neurons that were positive for glutamate also expressed the 5-HT_1B_ (64%), 5-HT_1D_ (68%), and the 5-HT_1F_ (60%) receptor [79]. These three receptors are situated such that they can modulate the release of glutamate from CNS terminals of the TG neurons. Glutamate has been shown to mediate trigeminovascular nociceptive transmission through N-methyl-D-aspartate (NMDA) and non-NMDA receptors in the TNC [49], and it is an important neurotransmitter in the development and maintenance of central sensitization. Thus, inhibition of glutamate release in the TNC via 5-HT receptor activation may not only attenuate early stages of migraine headache, but may reduce glutamate effects during the later stages where central sensitization and cutaneous allodynia are believed to occur [31, 33–36] . Double-labeling studies performed in the vestibular nuclei of rats revealed that neurons positive for glutamate [80] or CGRP [81] also expressed label for the 5-HT_1F_ receptor, providing additional evidence that activation of this receptor may inhibit the release of glutamate and CGRP in CNS sites.

Imaging studies have implicated several brain regions in migraine, including the thalamus PAG, and the TNC [24, 82]. Expression of 5-HT_1F_ receptors in the thalamus represents another site where a 5-HT_1F_ agonist might contribute to an anti-migraine effect, possibly in part by preventing or reversing central sensitization; however, this has not been confirmed or refuted by any studies. The midbrain PAG is a region that is intricately linked to analgesia, and also may contribute to migraine [83]. Early clinical studies with electrical stimulation of the PAG produced relief of otherwise intractable pain [84]. Unexpectedly, some patients also developed migraine-like headaches after implantation of the electrodes into the PAG [84]. fMRI studies have shown activation of the PAG during a migraine attack. The microinjection into the PAG of naratriptan, which has equivalent affinity for the 5-HT_1B_, 5-HT_1D_, and 5-HT_1F_ receptors [52], produced a seemingly selective inhibition of TNC neurons to meningeal stimuli, but not cutaneous facial stimuli [85]. In that study, the evoked responses of TNC neurons were recorded in response to stimuli applied to the dura, facial skin, and cornea. Microinjection of naratriptan into the PAG inhibited nociceptive inputs in the TNC from dural, but not facial or corneal nociceptive stimuli [85]. Although this study focused on the potential role of 5-HT_1B/1D_ receptors in modulating TNC neurons from the PAG, the potential role of 5-HT_1F_ receptors was not excluded.

Migraine involves activation of PNS nerve endings that send signals from the dural meninges covering the brain to the TG; then the signal is propagated to CNS brain stem nuclei such as the trigeminal nucleus caudalis, followed by signaling to the hypothalamus and thalamus and ascending signals to the cortex (Fig. 1a,b,c) [72–76, 82]. When serotonin binds to the 5-HT_1F_ receptor, it inhibits neuropeptide and neurotransmitter release, inhibiting transmitter local activity and downstream neuronal signaling (Fig. 2b, Fig. 1d) [74, 75, 81, 86].

## Lasmiditan mechanism of action

### 5-HT_1F_ agonists similar to Lasmiditan

Compounds LY344864 and LY334370 are selective ligands for the 5-HT_1F_ receptor that were not commercialized as clinical therapies but are useful pharmacologic tools for the characterization of this receptor. LY344864 binds to the cloned human 5-HT_1F_ receptor with a K_i_ of 6 nM, and with poor affinity (≥500 nM) for other serotonergic receptors [87]. LY344864 inhibited forskolin-induced accumulation of cAMP and inhibited plasma extravasation in rats [87]. Whereas LY344864 did not produce any contractions of rabbit saphenous vein up to a concentration of 10^− 4^ M, the triptans: sumatriptan, zolmitriptan, rizatriptan, and naratriptan, all produced contractions, suggesting vasoconstrictive activity [9]. CNS penetration of a single peripheral dose of LY344864 (1 mg/kg, IV) was indicated by persistent cortical levels detected over 8 h [87]. Systemically administered LY344864 inhibited c-*fos* expression in the TNC of rats in response to intracisternal administration of capsaicin [88]. Increased expression of the immediate early gene *c-fos* or of its protein product Fos is a reliable biomarker of nociceptive stimulation, while inhibition of *c-fos* expression and TNC neuronal activity are suggestive of a CNS-mediated effect [52].

LY334370 is also a highly selective agonist for the 5-HT_1F_ receptor, with a K_D_ of 0.446 nM for the human 5-HT_1F_ receptor [89]. There was a statistically significant correlation between [^35^S] GTP binding of LY334370 in cell homogenates expressing the 5-HT_1F_ receptor and forskolin-stimulated cAMP formation as well as with plasma protein extravasation caused by electrical stimulation of meninges [89, 90]. In addition, LY334370 inhibited Fos expression in the TNC and reduced neuronal firing rates of TNC neurons in response to dural stimuli, suggesting a potential CNS-mediated effect [52, 88]. LY334370 did not induce contractions in the rabbit saphenous vein either alone or in the presence of a baseline vascular tone induced by PGF_2α_ [52], and LY334370 did not constrict human meningeal and cerebral arteries in vitro [91]. This compound was found to be effective and well tolerated for the acute treatment of episodic migraine [52, 91]. Adverse events (AE) included asthenia, dizziness, and somnolence, which suggest that LY334370 acts at CNS sites. It was not commercially developed, however, as preclinical toxicology results suggested potential off-target liver toxicity [52].

#### Lasmiditan

Lasmiditan (formerly COL-144, LY573144) is a first-in-class “ditan” compound that has a pyridinoyl-piperidine scaffold and differs from LY334370 and triptans by the absence of an indole core [92]. Lasmiditan is a selective 5-HT_1F_ agonist approved in the US for the acute treatment of migraine with or without aura in adults [93]. It is highly selective for the human 5-HT_1F_ receptor, with a K_i_ of 2.21 nM, compared to values of 1053 nM, 1043 nM, and 1357 nM for the 5-HT_1A/B/D_ receptors, respectively [92]. Lasmiditan has greater than 440 times more potent binding affinity for 5-HT_1F_ versus 5-HT_1B_ and 5-HT_1D_ receptors. Lasmiditan showed no discernable agonist activity at the 5-HT_1B/D_ receptors as determined by stimulation of [^35^S]-GTPγS binding, but had nanomolar efficacy at the 5-HT_1F_ receptor in this assay [92]. In a recent study where second-messenger activity of receptor activation was analyzed, it was found that almotriptan, avitriptan, eletriptan, frovatriptan, naratriptan, sumatriptan, and zolmitriptan showed varying magnitudes of agonist activity at the 5-HT_1B/D_ as well as the 5-HT_1F_ receptors, whereas lasmiditan only showed agonist activity at the 5-HT_1F_ receptor [94].

In studies performed with several structurally diverse serotonergic agonists, there was a significant correlation between contractile potency in either the rabbit or canine saphenous vein with potency for contraction of human cerebral arteries, suggesting that the saphenous vein assay may be predictive of human vasoconstrictive activity [95]. Lasmiditan has no discernable vasoconstrictive effects at concentrations up to 100 μM in the rabbit saphenous ring assay, which is a reliable predictor of human coronary artery vasoconstrictor liability [92]. More recently, the activity of lasmiditan in isolated human coronary arteries, internal mammary arteries, and middle meningeal arteries was compared to that of sumatriptan [94]. Lasmiditan did not produce any significant contractions at all doses tested in these isolated human arteries, whereas dose-dependent contractions occurred with sumatriptan. In in vivo studies in anesthetized beagles, lasmiditan did not decrease carotid artery diameter or blood flow at clinically relevant doses [94]. Activation of second messenger for the 5-HT_1B_ receptor, but not for the 5-HT_1D_ or the 5-HT_1F_ receptors, correlated significantly with contractile potency [94]. These in vitro human isolated blood vessel and in vivo anesthetized canine results suggest that, unlike triptans, lasmiditan is not vasoconstrictive at active doses.

In one study, dilation of the middle meningeal artery in response to endogenously released CGRP via either capsaicin injection or periarterial electrical stimulation, as well as to exogenously administered CGRP, was measured in anesthetized rats [96]. Lasmiditan (0.3–10 mg/kg) or sumatriptan (3–10 mg/kg) produced significant (*p* < 0.05) dose-dependent inhibition of endogenous but not exogenous CGRP, indicating that lasmiditan can inhibit dural CGRP release but is not a CGRP receptor antagonist [96]. Unlike sumatriptan, lasmiditan did not produce any significant blood pressure changes [96]. In other studies, lasmiditan blocked electrical stimulation of trigeminal afferent neurons innervating the dura activated TNC neurons [76]. Lasmiditan that was orally administered 1 h prior to electrical stimulation of the TG of anesthetized rats inhibited dural plasma protein extravasation in a dose-dependent manner [92]. In addition, orally-administered doses of lasmiditan blocked the expression of *c-fos* in the TNC in response to electrical stimulation of the TG [92]. Together with the absence of vasoconstrictive effect, these preclinical results showed that lasmiditan has high potency and efficacy at the 5-HT_1F_ receptor, but not the 5-HT_1B_ or 5-HT_1D_ receptors.

The induced expression of Fos in the TNC is a marker of nociceptive activation of the second-order neurons, and is mediated largely through glutamate released from trigeminal nerve terminals in the TNC [97]. Moreover, glutamate release drives central sensitization in the TNC [98]. Consequently, lasmiditan may alleviate migraine at least in part by activation of 5-HT_1F_ receptors present on glutaminergic trigeminal nerve terminals in the TNC [79].

RCTs have demonstrated that lasmiditan is efficacious, safe, and generally well tolerated for the acute treatment of migraine. In a proof-of-concept, multi-center trial (NCT00384774), intravenous (IV) doses of 10 mg to 40 mg of lasmiditan resulted in pain-free rates at 2 h ranging from 20.8% (10 mg) to 37.5% (30 mg), compared to a 19% rate for placebo [99]. The response rates, based on patients reporting reduction in headache severity from moderate or severe at baseline to mild or no headache, were 54% to 75% at 2 h, which were significantly (*p* = 0.013) superior to placebo [99]. AEs included dizziness, paresthesia, and sensations of heaviness, suggestive of CNS-mediated effects [99].

In another phase II RCT (NCT00883051), oral doses of 50, 100, 200, or 400 mg of lasmiditan likewise proved significantly superior to placebo in reducing migraine headache severity from moderate or severe at baseline to mild or no headache at 2 h [12]. A significantly greater proportion of patients was pain-free at 2 h after 200 mg (19%, *p* = 0.032) and 400 mg (28%, *p* = 0.0007) of lasmiditan [12].

SAMURAI (NCT02439320), a phase III RCT, evaluated the efficacy of oral doses of lasmiditan (100 and 200 mg) [100] and demonstrated that a significantly (*p* < 0.001) greater proportion of patients was headache-free at 2 h with 100 mg (28.2%) and 200 mg (32.2%) mg of lasmiditan, compared to placebo (15.3%) [100]. Similar results were obtained with the phase III RCT SPARTAN (NCT02605174), but this study did not exclude patients who had coronary artery disease, cardiac arrhythmias, or uncontrolled hypertension [13]. The presence of cardiovascular risk factors in patients receiving lasmiditan did not result in either decreased efficacy of lasmiditan or in an increase in frequency of likely cardiovascular treatment-emergent AEs [101]. Overall, the most commonly reported AEs in both phase III studies and in the phase II study with the orally-administered lasmiditan were dizziness, paresthesia, somnolence, fatigue, nausea, lethargy (including a sensation of heaviness), and vertigo [102].

The AEs of somnolence and fatigue suggest that lasmiditan produces CNS-mediated effects [102, 103]. AEs such as dizziness and vertigo associated with lasmiditan could be generated through PNS-mediated effects since vestibular organelles (e.g., cochlear inner hair cells and vestibular hair cells) and the vestibular ganglion are extensively positive for 5-HT_1F_ receptor immunoreactivity [63]. PNS vestibular serotonergic activity is likely associated with comorbidity of migraine and balance disorders, vestibular migraine, and migraine-related tinnitus or phonophobia [63, 65, 104, 105]. This concept is supported by the fact that IV serotonin infusions can elicit plasma extravasation in specific inner ear tissues, vestibular nerve, and associated ganglia [106].

#### Comparison of Ditans with Triptans

Although triptans are considered the gold standard for the treatment of acute migraine, they still present some drawbacks [74, 107]. Their mechanism is mediated by the 5-HT_1B_ receptor and includes vasoconstriction, so they are contraindicated in patients who have cardiovascular or cerebrovascular disease [75]. Moreover, triptans are ineffective in approximately 40% of migraine attacks and provide insufficient efficacy for about one-quarter of patients [74, 107].

Ditans provide a different mechanism of action compared to triptans in the treatment of a migraine attack. A post-hoc analysis of pooled data from the phase III SAMURAI and SPARTAN RCTs was undertaken to determine whether the efficacy of lasmiditan differed because of prior triptan therapy response [108]. Regardless of prior response to triptan use, patients taking lasmiditan had greater efficacy than those taking placebo. This benefit did not vary significantly between patients with a good response and those with an insufficient response (i.e., insufficient efficacy) to prior triptan use. Treatment-emergent AEs were similar across subpopulations regardless of prior triptan experience or response to triptan use [108].

It is unclear if there is a CNS component to the efficacy of triptans against migraine. Sumatriptan is poorly lipophilic and does not cross the BBB to any appreciable extent. CNS effects may contribute to the efficacy of second-generation triptans (e.g. zolmitriptan, naratriptan, rizatriptan, almotriptan, eletriptan, frovatriptan, donitriptan, and avitriptan) [74]. However, literature values for triptans’ distribution coefficients, indicative of lipophilicity, vary considerably. Although these triptans are somewhat more lipophilic than sumatriptan, their values do not differ much, and their ability to penetrate the CNS is poor when compared to CNS-active drugs [74, 109, 110].

BBB efflux transporters such as P-glycoprotein (P-gp) can substantially reduce the CNS concentration of triptans [74, 111]. For example, the brain concentration of eletriptan, the most lipophilic among the triptans, was 40 times greater in knock-out mice that did not express the *Mdr1a* gene, which codes for P-gp, when compared to the wild type; thus, eletriptan is considered to be a “good” substrate for this efflux transporter [74, 111]. The brain:blood ratios of naratriptan and rizatriptan were significantly reduced in mice expressing *Mdr1a*, suggesting that they also may be efflux transporter substrates [111]. Other studies have concluded that there is no significant correlation between lipophilicity coefficients of triptans and their efficacy in relieving migraine [110]. These data suggest there may not be a major CNS mechanism associated with triptans’ therapeutic effect in migraine. Of note, neither the small molecule CGRP antagonists, nor the large monoclonal antibodies [57] appear to have any clinically appreciable ability to cross the BBB; thus, they may also not have a CNS component to their efficacy.

Lasmiditan, on the other hand, is highly lipophilic and capable of penetrating the BBB (Fig. 1e, Supplemental Video) [10, 92, 112]. Studies with epithelial cell lines revealed that lasmiditan had significant permeability across cell membranes and thus would readily cross the BBB [10]. In a preclinical study with mice receiving an IV injection of 1 mg/kg of lasmiditan, the brain/plasma ratio 2 h after injection was 1.57, indicating a high degree of CNS penetration for lasmiditan [10]. Lasmiditan crossed the BBB of rats as evidenced by brain-to-plasma ratios after IV and oral dosing, with CNS lasmiditan concentration of 0.7 to 1.2 μg Eq/g vs. 0.9 μg Eq/g in the blood/plasma [10]. These preclinical in vitro and in vivo animal distribution observations indicated that lasmiditan can cross the BBB and potentially alleviate migraine through CNS mechanisms, possibly in combination with PNS effects (Fig. 1e,f, Supplemental Video) [10, 92, 112]. This is consistent with the fact that the most common AEs associated with lasmiditan are CNS-mediated effects [102, 103]. However, the fact that lasmiditan has some central effects does not prove that its efficacy against migraine is mediated in the CNS. Preclinical and ex vivo evidence suggested that lasmiditan may inhibit PNS and CNS pain pathways, including the trigeminal nerve. Inhibition is likely via decreased release of neuropeptides and neurotransmitters such as CGRP and glutamate, thereby attenuating local activity and neuronal firing (Fig. 1e,f) [75].

#### A role for mitochondria?

It has been suggested that neuronal excitability and susceptibility to migraine could be due in part to mitochondrial dysfunction [113]. Studies using phosphorus magnetic resonance spectroscopy showed impaired brain oxidative energy metabolism related to mitochondrial dysfunction in patients with migraine between and during attacks [114, 115]. Conversely, a double-blind RCT reported that a proprietary blend of Mg^++^, coenzyme Q10 (designed to enhance mitochondrial function), and riboflavin significantly reduced the burden of migraine [116]. Epigenetic regulation of mitochondrial DNA could represent a factor in the migraine pathogenesis [113].

Mitochondrial biogenesis helps maintain mitochondrial homeostasis, and the induction of mitochondrial biogenesis has been shown to normalize mitochondrial dysfunction [117]. In a series of in vitro and in vivo studies, it was found that activation of 5-HT_1F_ receptors with the selective agonists LY334370 and LY344864 increased carbonyl cyanide-p-trifluoromethoxyphenylhydrazone–uncoupled oxygen consumption in renal proximal tubule cells and increased the expression of mitochondrial proteins [117]. Small interfering RNA knockdown of the 5-HT_1F_ receptor blocked mitochondrial biogenesis induced by LY334370 and LY344864. Moreover, LY344864 enhanced the recovery of renal function in an animal model of acute kidney injury by increasing mitochondrial biogenesis [117]. In other studies, LY344864 enhanced mitochondrial homeostasis in control and in 6-hydroxydopamine lesioned mice, and increased locomotor activity in the lesioned mice [118]. These data suggest mitochondrial dysfunction could have a role in migraine pathophysiology, and that perhaps 5-HT_1F_ receptor activity could help offset this dysfunction.

Treatment of human glomerular endothelial cells and mouse glomerular endothelial cells with 5-HT_1F_ receptor agonists LY344864 or lasmiditan (0–500 nM) induced mitochondrial biogenesis as evidenced by maximal mitochondrial respiration, increased nuclear- and mitochondrial-encoded proteins, and increased mitochondrial number [119]. Collectively these studies showed that agonists at the 5-HT_1F_ receptor can enhance mitochondrial activity. The role mitochondrial dysfunction may play in migraine pathophysiology requires further investigation. Whether or not restoration of normal mitochondrial function through 5-HT_1F_ receptor activity plays a role in treatment for migraine also needs additional research.

## Conclusion

The discovery that activation of 5-HT_1F_ receptors could provide benefits without the cardiovascular risks associated with triptans was an important advancement in the research on therapeutics for the treatment of migraine. Evidence suggested lasmiditan PNS activation of the 5-HT_1F_ receptor can inhibit the release of CGRP from trigeminal afferent nerves in the PNS [63], thus mimicking the behavior of triptans, CGRP antagonists, and CGRP antibodies. Lasmiditan appears to cross the BBB and may also act upon 5-HT_1F_ receptors found in CNS sites where the receptor potentially can modulate central sensitization of the TNC and thalamus. Receptor activation may also activate elements of descending pain modulation. Lasmiditan activation of the 5-HT_1F_ receptor does not mediate vasoconstriction, thus eliminating a risk associated with triptans. Evidence is emerging that agonists at the 5-HT_1F_ receptor may also stimulate mitochondrial biogenesis, which potentially could help mitigate migraine, although more research is needed.

Lasmiditan is a highly selective, lipophilic, high-affinity 5-HT_1F_ agonist. While the precise mechanism of action is unknown, preclinical in vitro and in vivo animal data as well as ex vivo human evidence suggested that lasmiditan exerts its therapeutic effects through agonist action at PNS and CNS 5-HT_1F_ receptors. This profile suggested that lasmiditan may act through a mechanism not fully engaged by current migraine therapies. Lasmiditan may be beneficial for patients who have insufficient efficacy, a contraindication, or an intolerability to other current migraine therapeutics.

## Data Availability

The literature and datasets used for the current study are available from the corresponding author on reasonable request.
